# Engineering interface-type resistive switching in BiFeO_3_ thin film switches by Ti implantation of bottom electrodes

**DOI:** 10.1038/srep18623

**Published:** 2015-12-22

**Authors:** Tiangui You, Xin Ou, Gang Niu, Florian Bärwolf, Guodong Li, Nan Du, Danilo Bürger, Ilona Skorupa, Qi Jia, Wenjie Yu, Xi Wang, Oliver G. Schmidt, Heidemarie Schmidt

**Affiliations:** 1 State Key Laboratory of Functional Material for Informatics, Shanghai Institute of Microsystem and Information Technology, Chinese Academy of Sciences, Shanghai 20050, P. R. China; 2 Material Systems for Nanoelectronics, Technische Universität Chemnitz, Chemnitz 09126, Germany; 3 IHP, Im Technologiepark 25, Frankfurt (Oder) 15236, Germany; 4 Institute for Integrative Nanosciences, IFW Dresden, Dresden 01069, Germany; 5 HZDR Innovation GmbH, Dresden 01328, Germany

## Abstract

BiFeO_3_ based MIM structures with Ti-implanted Pt bottom electrodes and Au top electrodes have been fabricated on Sapphire substrates. The resulting metal-insulator-metal (MIM) structures show bipolar resistive switching without an electroforming process. It is evidenced that during the BiFeO_3_ thin film growth Ti diffuses into the BiFeO_3_ layer. The diffused Ti effectively traps and releases oxygen vacancies and consequently stabilizes the resistive switching in BiFeO_3_ MIM structures. Therefore, using Ti implantation of the bottom electrode, the retention performance can be greatly improved with increasing Ti fluence. For the used raster-scanned Ti implantation the lateral Ti distribution is not homogeneous enough and endurance slightly degrades with Ti fluence. The local resistive switching investigated by current sensing atomic force microscopy suggests the capability of down-scaling the resistive switching cell to one BiFeO_3_ grain size by local Ti implantation of the bottom electrode.

Nonvolatile resistive switching has been observed in many material systems within a simple structure consisting only of a metal-insulator (or semiconductor)-metal (MIM) sandwich stack.^1^ The engineering of resistive switching has attracted great attention due to the potential application of MIM structures in the next generation of nonvolatile memory^1^,^2^,^3^, reconfigurable nonvolatile logics^4^,^5^ and data encryption^6^. Recent intensive investigations have provided a deep understanding of the resistive switching mechanisms in oxides. There is a general agreement on the role of the ion migration (oxygen vacancies migration) within an applied electric field, which induces the formation and rupture of conductive filaments inside the filamentary-type oxide resistive switches^7^,^8^,^9^,^10^, and which induces a reversible change of the barrier heights at the interfaces inside interface-type oxide resistive switches^5^,^11^,^12^,^13^,^14^. It is reported that the ion migration is related to the thermal diffusion of the metallic atoms from the active bottom/top metallic electrodes, e.g. Cu^15^,^16^, Ag^16^,^17^, Ni^18^, and Al^19^, or even from the adhesion layer under the bottom electrodes, e.g. Ti^20^,^21^,^22^. The diffused metallic atoms can be oxidized and incorporated into the oxide thin films^23^, and the created cations can either induce oxygen vacancies which are mobile in the oxide thin films^20^,^21^,^22^ or itself migrate within an applied electric field^15^,^16^,^17^,^18^,^19^. In other words, the diffused metallic atoms seed the nanoscale switching centers in the resistive switches^20^. However, technically, the metallic diffusion from the bottom electrodes or the adhesion layer is often poorly controlled and restrains the options of metallic materials used for the bottom electrodes. In addition, the metallic diffusion often occurs over the whole wafer chip which is in contradiction with the complementary metal-oxide-semiconductor (CMOS)-compatible technology.

BiFeO_3_ (BFO) has been intensively investigated as an oxide resistive switch^24^,^25^,^26^,^27^,^28^,^29^ thanks to its fascinating physical properties, e.g., ferroelectric effect and photovoltaic effect^30^,^31^, which offers the possibility to integrate multi-functionality into one single device. In our previous works^2^,^32^,^33^,^34^,^35^,^36^, BFO thin films on Pt/Ti/Sapphire or Pt/Ti/SiO_2_/Si substrates show excellent bipolar resistive switching performances such as electroforming free, multi-level states, long retention time, and stable endurance, in which the Ti diffusion from the bottom electrodes during BFO thin film deposition plays an important role. A model based on modifiable Schottky barrier heights was proposed to explain the bipolar resistive switching in BFO thin films, in which the ionized oxygen vacancies (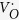) and diffused Ti act as mobile and fixed donors, respectively. The mobile 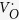 donors are redistributed by a writing bias which changes the Schottky barrier height at the bottom interface, and the fixed Ti donors can trap the mobile 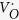 donors after the writing process to stabilize the resistive switching. In this work, we show that the Ti diffusion can be engineered before the BFO thin film deposition by Ti implantation of the Pt bottom electrode on Sapphire substrates. This offers a deeper understanding on the role of the fixed Ti donors in the resistive switching of BFO thin films.

## Methods

The Ti implantation of Pt(100 nm)/Sapphire substrates was carried out at room temperature with an ion energy of 40 keV and a series of Ti fluences. Subsequently, BFO thin films with a thickness of 460 nm were deposited on the Ti-implanted Pt/Sapphire substrates by pulsed laser deposition (PLD). For the PLD process, the nominal laser energy density, laser repetition rate, oxygen ambient pressure, and growth temperature were 2.6 J/cm^2^, 10 Hz, 0.013 mbar, and 650 °C, respectively. After the PLD process, the BFO thin films were *in-situ* annealed at 390 °C with the oxygen ambient pressure of 200 mbar for 60 minutes. Following the deposition, circular Au top contacts with an area of 0.045 mm^2^ and a thickness of 110 nm were prepared by DC magnetron sputtering at room temperature using a metal shadow mask. Thus, Au-BFO-Pt MIM structures with a series of Ti fluences were fabricated. The schematic sketches of the sample fabrication process and the sample structure are indicated in Figure S1 in supplementary information.

Electrical measurements were carried out using a Keithley source meter. For the time-of-flight secondary ion mass spectrometry (Tof-SIMS) measurements we used an IONTOF TOF-SIMS 5 equipment with an O_2_ sputtering beam (2000 eV) and a Bi analysis beam (25000 eV). The crater size was 300 μm × 300 μm. Atomic force microscopy (AFM) topography and current-sensing AFM (CsAFM) measurements were carried out with an Agilent Technologies 5420 Scanning Probe Microscope.

## Results and Discussions

### Ti distribution and morphologies of films

**Figure 1:**
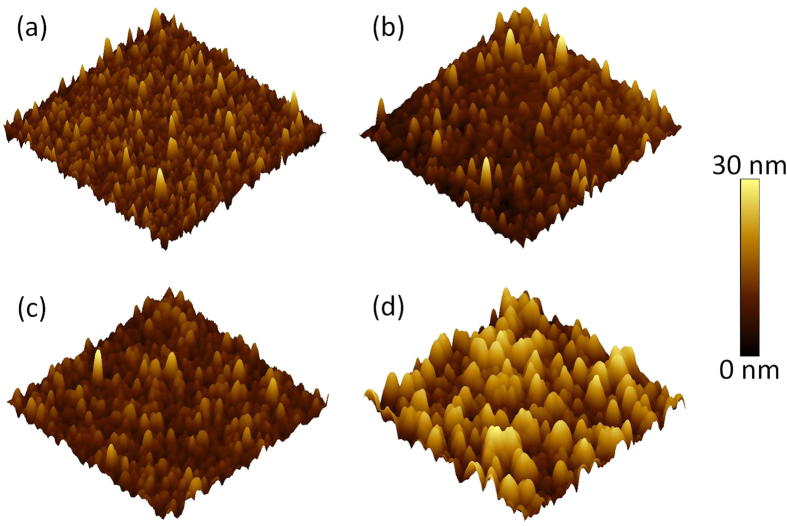
Three-dimensional AFM topography images of the pristine Pt/Sapphire (a) and the Ti-implanted Pt/Sapphire with Ti fluence of 5 × 10^15^ cm^−2^ (b), 1 × 10^16^ cm^−2^ (c), and 5 × 10^16^ cm^−2^ (d).The scanning size is 3 × 3 μm^2^. The mean arithmetic roughness (Ra) is 3.98 nm, 2.24 nm, 1.15 nm, and 4.99 nm, respectively. The AFM color scale (right side) indicates the height information.

The Ti distribution in the Pt/Sapphire after the Ti implantation was estimated by the Stopping and Range of Ions in Matter (SRIM) 2013 code^37^,^38^. The predicted concentrations of implanted Ti ions as a function of depth in Pt/Sapphire with different Ti fluences are shown in Figure S2 in supplementary information. It can be seen that Ti ions distribute within 50 nm below the surface of Pt layer and a concentration peak forms at the depth of ~10 nm. Note that SRIM as a static Monte Carlo program can only estimate the Ti distribution under the assumption that the initial stoichiometry of Pt/Sapphire is preserved. A sputtering yield of 9.36 for Pt was calculated by SRIM 2013, which suggests that around 15% of Pt atoms could be sputtered away at the Ti concentration peak with Ti fluence of 5 × 10^16^ cm^−2^. Experimentally, the Pt layer was completely removed when the Ti fluence was further increased, e.g. 1 × 10^17^ cm^−2^. The Ti implantation effect on the surface morphology of Pt/Sapphire was investigated by AFM as shown in Fig. 1. Pt grains with a typical size of 80 nm randomly distribute over the pristine Pt/Sapphire, and the mean arithmetic roughness (Ra) is 3.98 nm. After Ti implantation at low fluence of 5 × 10^15^ cm^−2^, the roughness is reduced to 2.24 nm. By further increasing the fluence to 1 × 10^16^ cm^−2^, the roughness is reduced to 1.15 nm, which may be due to the strain relaxation between the grains caused by the energy deposited by the implanted Ti ions. However, by further increasing the Ti fluence to 5 × 10^16^ cm^−2^, the typical Pt grain size dramatically increases to 170 nm, which may be due to the appearance of the disordering induced agglomeration of grains. Consequently, the roughness is increased to 4.99 nm. A comparable dependence of surface roughness on ion fluence has also been observed for ZnO thin films irradiated by Au ions^39^.

**Figure 2:**
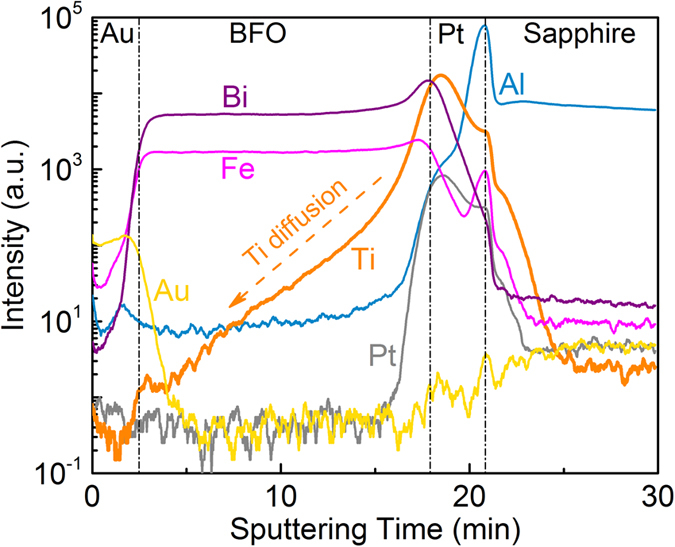
Tof-SIMS intensity-time profiles of the metallic elements in the MIM structure with Ti fluence of 5 × 10^16^ cm^−2^.

It is expected that the Ti migration into BFO layer is more efficient along the BFO grain boundaries and occurs during the PLD process at 650 °C^40^. Figure 2 shows the Tof-SIMS intensity-time profiles of the BFO thin film on Ti-implanted Pt/Sapphire with Ti fluence of 5 × 10^16^ cm^−2^, depicting Au, Bi, Fe, Ti, Pt, and Al ion intensities as a function of sputtering time. It is clear that the Ti intensity profile exhibits a broader shoulder compared to that of other metallic elements, which indicates that Ti diffused into the BFO thin films during the PLD process and that a Ti concentration gradient was created along the BFO growth direction. The Ti diffusion into BFO was also observed in the BFO thin films on Pt/Ti/Sapphire or Pt/Ti/SiO_2_/Si substrates in our previous works, which plays a crucial role for the resistive switching in BFO thin films^36^,^40^. In these MIM structures, Pt layer serves not only as a bottom electrode but also as a diffusion suppressing layer to prevent a strong Ti diffusion into BFO layer during the BFO deposition at 650 °C. Therefore, an optimized concentration of fixed Ti donors is realized and a tunable Schottky barrier can be formed at the BFO/Pt/Ti interfaces. It is expected that less Ti diffuses into the BFO layer in MIM structures with Pt bottom electrodes which have been implanted with a smaller Ti fluence. The surface morphology of the BFO thin films on Ti-implanted Pt/Sapphire was characterized by AFM measurements with a scanning size of 3 × 3 μm^2^ (Supplementary information, Figure S3). The mean arithmetic surface roughness of BFO thin films is 12.5 nm, 9.54 nm, and 13.1 nm for the Ti fluence of 5 × 10^15^ cm^−2^, 1 × 10^16^ cm^−2^, and 5 × 10^16^ cm^−2^, respectively.

### Resistive switching characteristics

**Figure 3:**
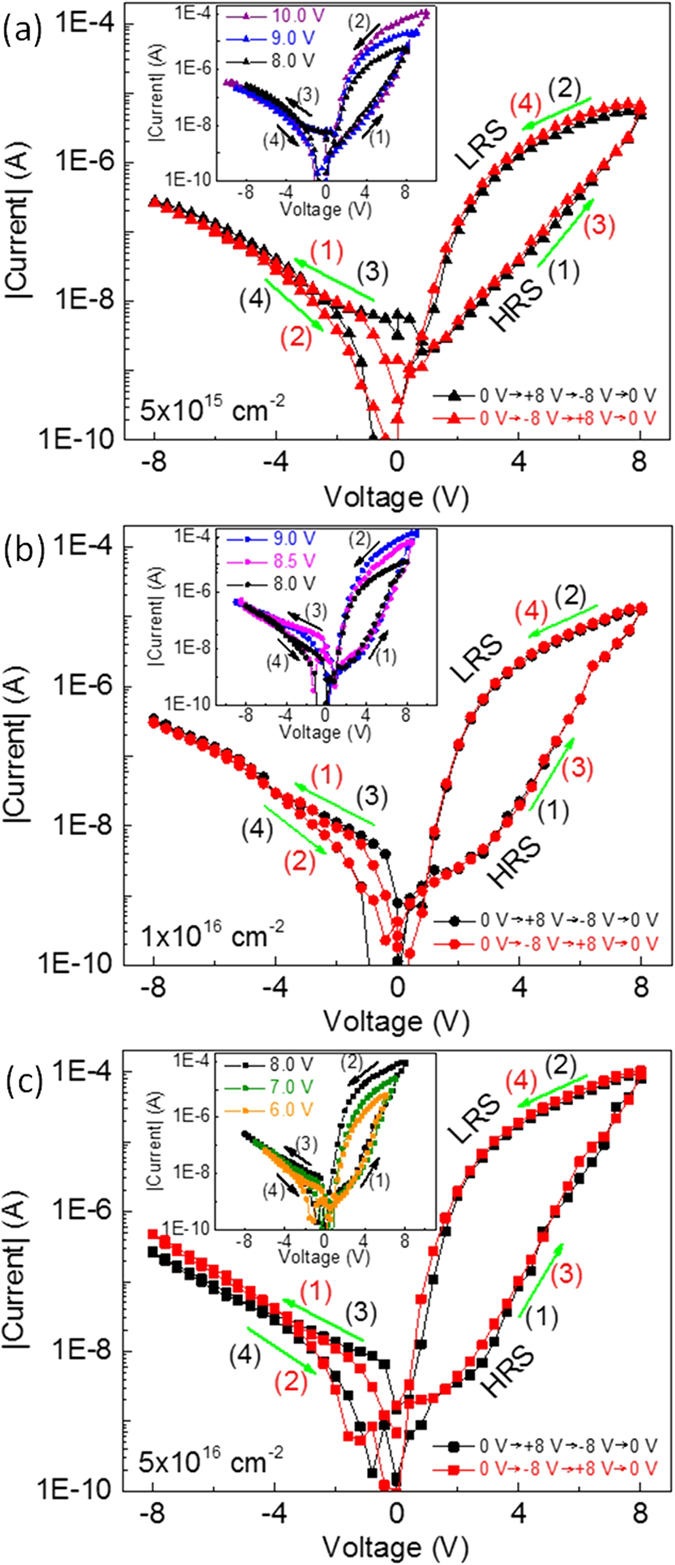
Typical I-V characteristics with different voltage sweeping sequences measured at two pristine cells on the MIM structures with Ti fluence of 5 × 10^15^ cm^−2^ (a), 1 × 10^16^ cm^−2^ (b), and 5 × 10^16^ cm^−2^ (c).The insets show the I-V characteristics with the same voltage sweeping sequences but different maximum voltage measured at the same cell on the MIM structures. Note that to avoid a hard breakdown of the devices, the maximum current was limited to be 100 μA. The sets of the maximum voltages are [8.0 V, 9.0 V, 10.0 V], [8.0 V, 8.5 V, 9.0 V] and [6.0 V, 7.0 V, 8.0 V] for the MIM structures with Ti fluence of 5 × 10^15^ cm^−2^, 1 × 10^16^ cm^−2^, and 5 × 10^16^ cm^−2^, respectively. The numbers (1)–(4) and the arrows indicate the voltage sweeping sequences and the voltage sweeping directions, respectively.

The current-voltage (I-V) measurements were carried out with a Keithley 2400 source meter. The schematic sketch of the electrical measurement configuration is indicated in Figure S1 in supplementary information, in which the bias voltage was applied on Au top electrode and the Ti-implanted Pt bottom electrode was grounded. As shown in Fig. 3, the shape of the I-V characteristics obtained from the BFO on Ti-implanted Pt/Sapphire is quite similar to that reported in our previous works^2^,^32^,^33^,^34^,^35^,^36^,^40^. The I-V characteristics were measured by sweeping the voltage in sequence of 0 V → +8 V → −8 V → 0 V (black curve) and 0 V → −8 V → +8 V → 0 V (red curve) on two pristine cells of the MIM structures, respectively. In both cases, a distinct I-V hysteresis exists in the positive bias range and no significant difference in the I-V characteristics is observed, which suggests that the electroforming process is not required for the resistive switching. Initially, the pristine MIM structures show high resistance state (HRS), and the low resistance state (LRS) is set by a positive bias while the HRS is reset by a negative bias. This indicates a bipolar resistive switching without an electroforming process for the MIM structures with different Ti fluences. Note that BFO thin films deposited on non-implanted Pt/Sapphire substrates do not show distinct resistive switching behavior (Supplementary information, Figure S4)^34^,^36^,^40^. Within the same applied bias range (between −8 V and +8 V) there is no obvious current difference in the negative bias range for the MIM structures with different Ti fluences, while the current in the positive bias range and the on/off current ratio at +2 V increase with increasing Ti fluence (Supplementary information, Figure S5).

The work function of Au and Pt amounts to 5.1 eV and 5.3 eV, respectively. The band gap of BFO is taken as 2.8 eV and the electron affinity is 3.3 eV^41^, and then the work function of n-type BFO should be less than 4.7 eV, which suggests an upward band bending in BFO at top Au/BFO and bottom BFO/Pt interfaces. Therefore, a Schottky barrier can be formed at both top and bottom interfaces. The observed resistive switching characteristics in BFO based MIM structures can be explained by a model of modifiable Schottky barrier height at bottom interface which can be tuned by the mobile 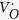 acting as the mobile donors (Supplementary information, Figure S6)^36^. With lower Ti fluence, a larger Schottky barrier height is expected to form at the bottom interface which will be discussed later. Therefore, a larger electric field is required to move the 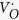 to the bottom interface to reduce the Schottky barrier height to fully set the structures to LRS. The insets in Fig. 3 show the I-V characteristics with different maximum voltages. The paths for the I-V curves in negative bias range (branches (3) and (4)) and the HRS branch of the I-V curve in positive bias range (branch (1)) are nearly the same which are independent of the maximum voltage. While the LRS branch of the I-V curve in positive bias range (branch (2)) are well separated from each other with different maximum voltage, indicating different LRS can be achieved depending on the applied maximum voltages. This multilevel LRS behavior offers an opportunity for designing multi-bit memories/logics^33^.

**Figure 4:**
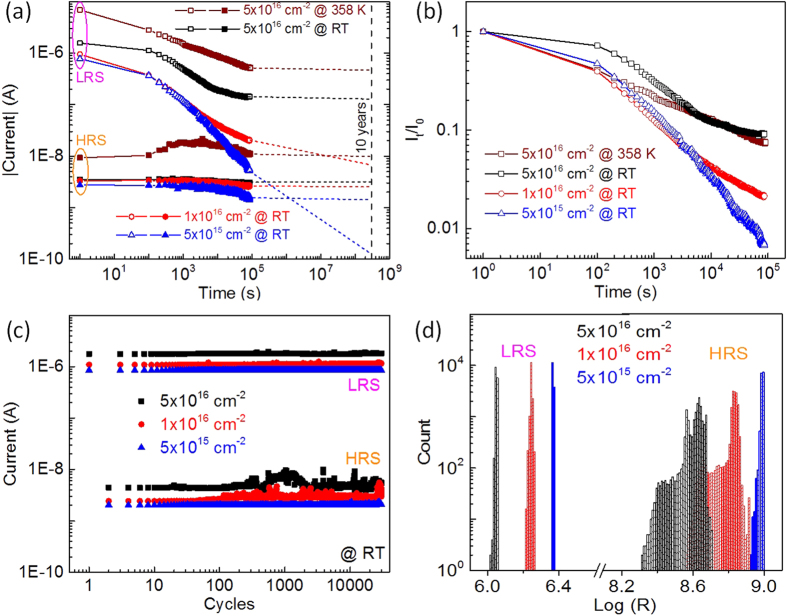
(**a**) Retention test results of the MIM structures with different Ti fluences. The extrapolated 10-years HRS/LRS retention time can be expressed by the dashed lines. (**b**) Normalized current of LRS vs. retention time. The current values taken at different time (I_t_) are normalized by the initially measured current value (I_0_). (**c**) Endurance test results of the MIM structures with different Ti fluences. (**d**) Statistics histograms of LRS/HRS in the endurance test results.

As shown in Fig. 4(a), the retention tests were carried out by first setting/resetting the MIM structures to LRS/HRS at room temperature, and then detecting the current with a small reading bias of +2 V every 2 min at room temperature (for the MIM structure with Ti fluence of 5 × 10^16^ cm^−2^, the current detection was performed at 358 K as well). In order to fully set/reset the MIM structures to LRS/HRS, the set/reset bias of +10 V/−10 V, +9 V/−9 V and +8 V/−8 V with pulse length of 100 ms were used for the MIM structures with Ti fluence of 5 × 10^15^ cm^−2^, 1 × 10^16^ cm^−2^, and 5 × 10^16^ cm^−2^, respectively. At room temperature, the HRS for all MIM structures are relatively stable, while degradation is observed during the LRS tests. The LRS of the MIM structures with low Ti fluences (both 5 × 10^15^ cm^−2^ and 1 × 10^16^ cm^−2^) decreases continuously during the retention tests, and the current ratio I_LRS_/I_HRS_ of the MIM decreases below 10 within 24 hours, while the LRS of the MIM structure with high Ti fluence (5 × 10^16^ cm^−2^) becomes stable after around 15 hours. The extrapolated current ratio I_LRS_/I_HRS_ can be well-kept at around 50 for more than 10 years as indicated by the dashed lines in Fig. 4(a). The change of LRS is more visible with the linear time axis (Supplementary information, Figure S7). Even at an elevated temperature of 358 K, the LRS of the MIM structure with Ti fluence of 5 × 10^16^ cm^−2^ can be stabilized within 24 hours and a current ratio I_LRS_/I_HRS_ larger than 30 can be obtained for more than 10 years. The HRS at elevated temperature initially exhibits a small decay. The similar effect was also observed in Au-BFO-Pt/Ti/Sapphire MIM structures, which was possibly due to the redistribution of 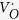 in HRS at elevated temperature^36^.

Figure 4(b) shows the normalized current in LRS, which suggests that the degradation in LRS becomes more pronounced with decreasing Ti fluence. In LRS the 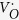 migrate to the bottom interface and then are trapped by the diffused Ti from the substrates during the BFO deposition, which consequently increases the doping concentration at the bottom interface and lowers the bottom Schottky barrier height^36^. The degradation in LRS is possibly due to the back diffusion of 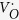 after the application of a positive writing voltage pulse which partially decreases the doping concentration at the bottom interface and partially starts to rebuild the bottom Schottky barrier. With lower Ti fluence, during the PLD process less Ti can diffuse from the hot Pt bottom electrode into the BFO layer. Therefore, not enough 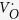 can be effectively trapped by Ti and the LRS is badly maintained (Supplementary information, Figure S6). The degradation of LRS in MIM structure with Ti fluence of 5 × 10^16^ cm^−2^ is stronger at an elevated temperature of 358 K because of the increasing diffusivity of 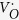 with increasing temperature. This suggests that a certain minimum amount of Ti in the BFO MIM structures is required to trap the mobile 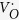 in the bottom interface in order to stabilize the resistive switching into LRS.

As shown in Fig. 4(c), the endurance tests were carried by repeating the process of set/read/reset/read for more than 3 × 10^4^ times at room temperature. Figure 4(d) shows the statistics histograms of the LRS/HRS in the endurance test results. In endurance tests, all MIM structures with different Ti fluences possess a relatively stable LRS and a narrow distribution of the resistance values in LRS. The relative fluctuation (standard deviation divided by mean value)^42^ of LRS is 0.20%, 0.91%, and 0.82% for the MIM structures with Ti fluence of 5 × 10^15^ cm^−2^, 1 × 10^16^ cm^−2^, and 5 × 10^16^ cm^−2^, respectively. However, the distribution of the resistance values in HRS is much broader than that in LRS. The relative fluctuation increases with the Ti fluences, i.e., 1.11%, 3.95%, and 12.34% for the MIM structures with a Ti fluence of 5 × 10^15^ cm^−2^, 1 × 10^16^ cm^−2^, and 5 × 10^16^ cm^−2^, respectively. The endurance can be improved by structuring the bottom electrodes or by local Ti implantation into the bottom electrodes^36^,^42^,^43^.

### Dependence of Schottky barrier height on the Ti fluence

The temperature-dependent I-V characteristics (from −2 V to +2 V) were measured by Keithley 2636A source meter (with a theoretical current resolution of 0.1 fA) after the MIM structures were fully set/reset to LRS/HRS (Supplementary information, Figure S8). The current increases with the temperature increasing from 253 K to 353 K. In HRS, the current is small in both positive and negative bias range showing head-to-head diode behavior as the Schottky-like barriers form at both top and bottom interface^36^. The reversed diode current can be governed by Poole-Frenkel emission^44^, Schottky emission^44^ or modified Schottky emission mechanisms^45^. The corresponding emission coefficients (Supplementary information, Figure S9-S10 and Table S1) suggest that electric conduction under reverse bias condition is consistent with the modified Schottky emission which is described by the modified Richardson-Schottky equation^45^:


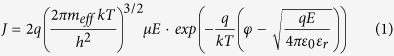


where *J*, *E*, *m*_*eff*_, *μ*, *φ*, and *ε*_*r*_ represent the current density, the electric field, the effective mass, the electron mobility, the potential barrier height, and the dielectric constant, respectively. The other symbols have their usual meaning. From equation (1), the Schottky-Simmons graphic representation can be obtained at a constant voltage (electric field) as follows:


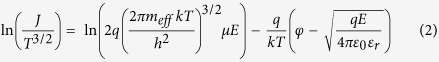


**Figure 5:**
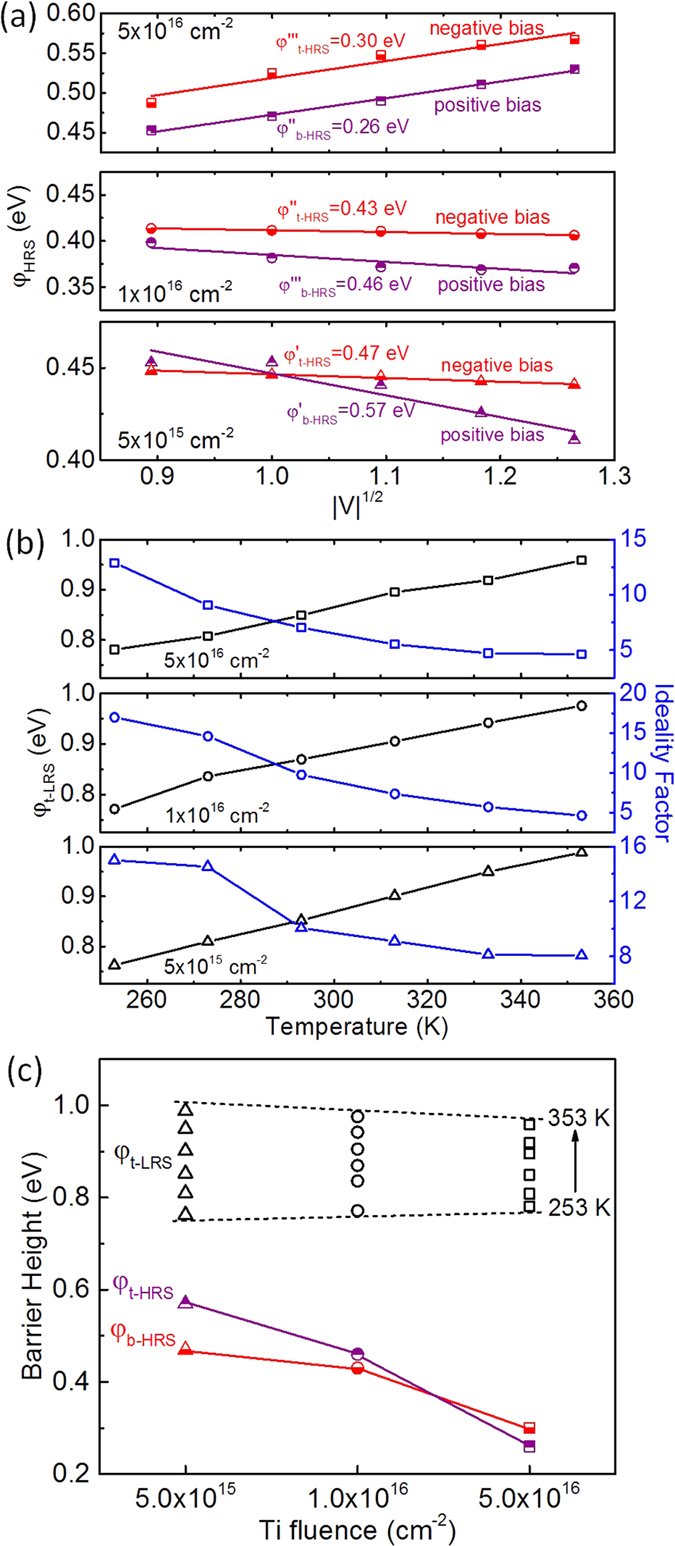
(**a**) Bias dependent Schottky barrier heights in HRS. The zero-bias Schottky barrier height can be extracted by a linear fitting. (**b**) Temperature dependent zero-bias Schottky barrier heights and ideality factors in LRS. (**c**) Change of the top and bottom Schottky barrier heights in LRS and HRS for the MIM structures with different Ti fluences.

The apparent potential barrier for the respective constant voltage (electric field) can be estimated from the slope of the representation of ln (J/T^3/2^)~1/T which gives a straight line. The temperature dependent I-V characteristics of the MIM structures with different Ti fluences were replotted in the Schottky-Simmons representation of ln (J/T^2/3^)~1/T at voltages of ±0.8 V, ±1.0 V, ±1.2 V, ±1.4 V and ±1.6 V. A linear fitting was obtained in both negative and positive bias range (Supplementary information, Figure S11). Figure 5(a) shows the apparent potential barrier height (φ_HRS_) calculated from the slope of the linear fitting in the plotting of ln (J/T^2/3^)~1/T as a function of |V|^1/2^. The barrier height at top interface can be obtained from the plotting in the negative voltages range (V<0 V) as the top Schottky barrier is reversed and dominates the current under the negative bias, and similarly the barrier height of reversed bottom Schottky barrier corresponds to the plotting in positive bias range (V>0 V). As shown in Fig. 5(a), with increasing reverse bias both top and bottom Schottky barrier heights decrease in the MIM structures with low Ti fluence (5 × 10^15^ cm^−2^ and 1 × 10^16^ cm^−2^), while the barrier heights of the MIM structure with Ti fluence of 5 × 10^16^ cm^−2^ increase. The Schottky barrier height depends on the doping concentration and the applied reverse bias, i.e. the Schottky barrier height decreases with increasing reversed bias in the case of low doping concentration but increases in the case of high doping concentration^46^. With larger Ti fluence, more Ti diffuses into BFO during the PLD process and the as-prepared BFO thin film possesses a higher doping concentration. Therefore, different changes of the Schottky barrier heights are presented. The potential barrier at zero bias can be extracted from the intercept of the linear fitting in the apparent potential barrier height as a function of |V|^1/2^. The top Schottky barrier height (*φ*_*t-HRS*_) is deduced to be 0.47 eV, 0.43 eV, and 0.30 eV for the MIM structures with Ti fluence of 5 × 10^15^ cm^−2^, 1 × 10^16^ cm^−2^, and 5 × 10^16^ cm^−2^, respectively, and the corresponding bottom one (*φ*_*b-HRS*_) is 0.57 eV, 0.46 eV and 0.26 eV, respectively.

In LRS, the I-V characteristics exhibit forwarded diode behavior due to the Schottky contact at top interface and Ohmic contact at bottom interface, and the current is mainly dominated by the Schottky barrier at top interface^36^. As shown in Fig. 5(b), the temperature dependent zero bias Schottky barrier height and ideality factor were fitted from the temperature dependent I-V curves by using the Shockley equation as follows:


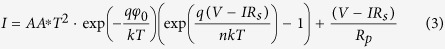


where *A*, A^*^, *φ*_*0*_, *R*_*s*_, *R*_*p*_ and *n* represent the area of the diodes, the effective Richardson constant, the zero bias barrier height, the series resistance, the parallel resistance and the ideality factor, and the other symbols have their usual meaning. The obtained Schottky barrier height (*φ*_*t-LRS*_) at the top interface decreases with the increasing temperature, while the ideality factor increases. The relatively large ideality factor may be due to the large series resistance in the order of several mega-ohm. By comparing the top and bottom Schottky barrier heights of the MIM structures in LRS and HRS as shown in Fig. 5(c), it is clear that the Schottky barrier height at top interface is greatly increased when the MIM structures are set to LRS as most of the 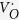 are drifted to the bottom interface, which is in agreement with the result in our previous report^36^. In HRS, the Schottky barrier height at both top and bottom interface (*φ*_*t-HRS*_ and *φ*_*b-HRS*_) decreases with the increasing Ti fluence because donors including the fixed Ti donors and mobile 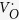 donors distribute relatively homogenously over the BFO layer in HRS^36^, and the Schottky barrier height is in inverse proportion to the doping concentration^12^,^13^. As the resulting Ti concentration in the BFO layer increases with the Ti fluence during Ti implantation into the underlying Pt bottom electrode, a larger Ti fluence causes a lower Schottky barrier height in HRS. However, as expected from the negligible Ti concentration close to the top electrode there is no significant difference between the top Schottky barrier height in LRS (*φ*_*t-LRS*_) for the MIM structures with different Ti fluence which varies between 0.76 eV and 0.99 eV. Most of the mobile 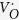 are drifted to the bottom interface to lower the bottom Schottky barrier height in LRS and the donor concentration at the top interface is very low. Thus, the top Schottky barrier height in LRS is independent of the Ti fluence. The relationship of the Schottky barrier height in LRS and HRS and the Ti fluence in turn is a good evidence for the model of modifiable Schottky barrier height for the resistive switching mechanism proposed in our previous publication^36^.

### Local Resistive Switching

**Figure 6:**
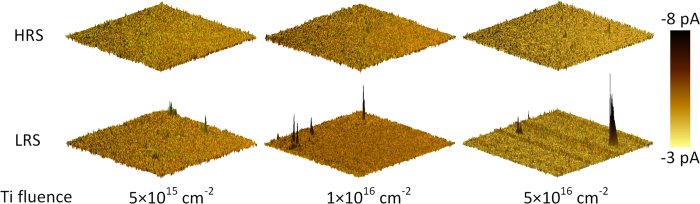
3D CsAFM current maps acquired with a reading bias of −4 V applied on the Pt bottom electrode for the MIM structures with different Ti fluences in HRS (upper) and LRS (lower).

The local resistive switching characteristics of the deposited BFO without Au top electrode were investigated by CsAFM measurements. A 3 × 3 μm^2^ large area on the BFO was switched to HRS and LRS by scanning a grounded conductive tip over the BFO surface while a constant voltage bias of +10 V and −10 V was applied to the Pt bottom electrode, respectively. Note that the voltage polarity is opposite to that in I-V measurements as indicated by the schematic sketch shown in Figure S1 in supplementary information. After that the current maps were measured by scanning the conductive tip over the same 3 × 3 μm^2^ large area with a small constant reading voltage of −4 V applied to the Pt bottom electrode as shown in Fig. 6. In HRS, only some small leakage current was detected which homogeneously distributes over the local area. Furthermore, there is no significant difference for the MIM structures with different Ti fluences. The maximum absolute value of the current in HRS is 5.79 nA, 5.64 nA and 5.50 nA with increasing Ti fluences. However, 2–4 conductive spots were observed in LRS. We expect that due to the nonuniform Ti distribution in BFO grains and BFO grain boundaries during the BFO deposition at 650 ^o^C and due to the nonuniform voltage drop over the polycrystalline BFO between CsAFM tip and large scale bottom electrode, the Schottky barrier height at bottom BFO/Pt/Ti interface is laterally inhomogeneous after scanning the CsAFM tip with writing bias. The current preferentially flows through the potential barrier minima and conductive spots can be observed^47^. Possibly, the highly resistive areas can be switched with a writing bias with larger magnitude or longer pulse length^36^. The maximum absolute value of the current in LRS increases with the increasing Ti fluences, i.e., 6.41 nA, 9.96 nA, and 12.81 nA for the MIM structures with Ti fluence of 5 × 10^15^ cm^−2^, 1 × 10^16^ cm^−2^, and 5 × 10^16^ cm^−2^, respectively. This is in agreement with the I-V characteristics and the calculated Schottky barrier heights. The local resistive switching suggests the possibility to scale down the nonvolatile resistive switching cell volume which is a function of the Ti distribution in the BFO thin films and of the film thickness dependent voltage drop.

### Conclusions

In conclusion, we have demonstrated the influence of Ti implantation of Pt bottom electrodes on Sapphire substrates on the resistive switching characteristics of the subsequently deposited BFO thin films. With decreasing Ti fluence the bottom Schottky barrier height increases, and a larger writing bias is required to fully set/reset the MIM structure. The retention performance can be improved while the endurance slightly degrades with increasing Ti fluence. This work provides a deeper understanding of the resistive switching in BFO thin film switches with a focus on the role of the diffused Ti. Resistive switching in BFO MIM structures can be engineered by Ti implantation of the bottom electrodes. Additionally, ion implantation as a microelectronic compatible process can be scaled down for generating the local resistive switching via defining a Ti pattern, which will allow to control the nonvolatile resistive switching cell volume in a CMOS/memristor hybrid chip.

## Additional Information

**How to cite this article**: You, T. *et al.* Engineering interface-type resistive switching in BiFeO_3_ thin film switches by Ti implantation of bottom electrodes. *Sci. Rep.* **5**, 18623; doi: 10.1038/srep18623 (2015).

## Supplementary Material

Supplementary Information
