# Supplementary material for: Engineering interface-type resistive switching in BiFeO3 thin film switches by Ti implantation of bottom electrodes
Source: Sci Rep. 2015 Dec 22;5:18623. doi: 10.1038/srep18623 (PMC4686890; doi:10.1038/srep18623)
Supplement: Supplementary Information [file srep18623-s1.pdf]

Supplementary Information

Engineering interface-type resistive switching in $BiFeO_3$ thin film switches by Ti implantation of bottom electrodes

Tiangui You,[1,2] Xin Ou,[1,*] Gang Niu,[3] Florian Bärwolf,[3] Guodong Li,[2,4] Nan Du,[2] Danilo Bürger,[2] Ilona Skorupa,[2,5] Qi Jia,[1] Wenjie Yu,[1] Xi Wang,[1] Oliver G. Schmidt,[2,4] Heidemarie Schmidt[2,*]

[1] State Key Laboratory of Functional Material for Informatics, Shanghai Institute of Microsystem and Information Technology, Chinese Academy of Sciences, Shanghai 20050, P. R. China

[2] Material Systems for Nanoelectronics, Technische Universität Chemnitz, Chemnitz 09126, Germany

[3] IHP, Im Technologiepark 25, Frankfurt (Oder) 15236, Germany

[4] Institute for Integrative Nanosciences, IFW Dresden, Dresden 01069, Germany

[5] HZDR Innovation GmbH, Dresden 01328, Germany

* Correspondence and requests for materials should be addressed to X.O. (ouxin@mail.sim.ac.cn), H.S (heidemarie.schmidt@etit.tu-chemnitz.de)

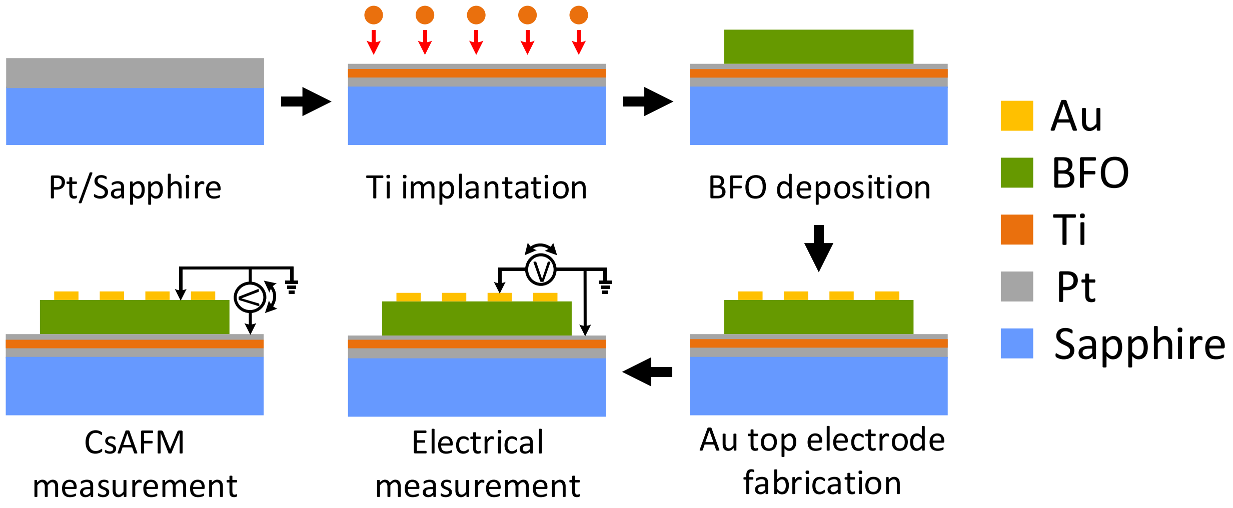

Figure S1. Schematic of the sample fabrication process and the measurement setups.

Figure S1 shows the schematic of the sample fabrication process and the measurement setups. The Ti implantation of Pt/Sapphire substrates was carried out at room temperature with an ion energy of 40 keV and a series of Ti fluences. After deposition of the BFO thin films on Ti-implanted Pt/Sapphire substrates by PLD, the Au top electrodes were prepared by DC magnetron sputtering at room temperature using a metal shadow mask. The electrical measurements were carried out with a Keithley source meter, and the bias voltage was applied on Au top electrodes while the Pt bottom electrode was grounded. However, in the CsAFM measurements, the bias voltage was applied to the Pt bottom electrode while the top conductive tip was grounded and scanned over the BFO surface.

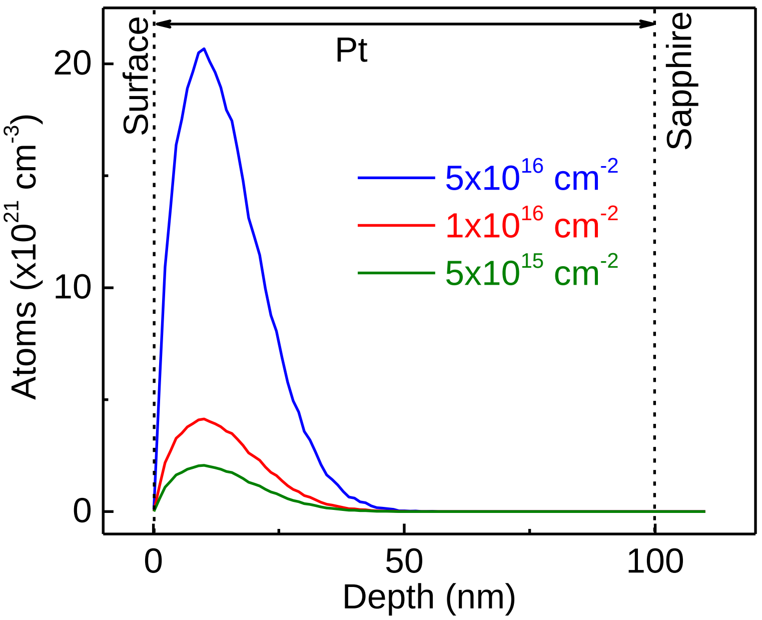

Figure S2. Calculated depth distribution of implanted Ti ions in Pt/Sapphire using SRIM.

Figure S2 shows the SRIM simulation results of the Ti distribution in the Pt/Sapphire after the Ti implantation. SRIM as a static Monte Carlo program can only estimate the Ti distribution under the assumption that the initial stoichiometry of Pt/Sapphire is preserved. It is expected that the Ti distributes within 50 nm below the surface of Pt layer and a concentration peak forms at a depth of ca. 10 nm. A sputtering yield of 9.36 for Pt was calculated by SRIM 2013, which suggests that a huge number of Pt atoms is sputtered during the Ti implantation process.

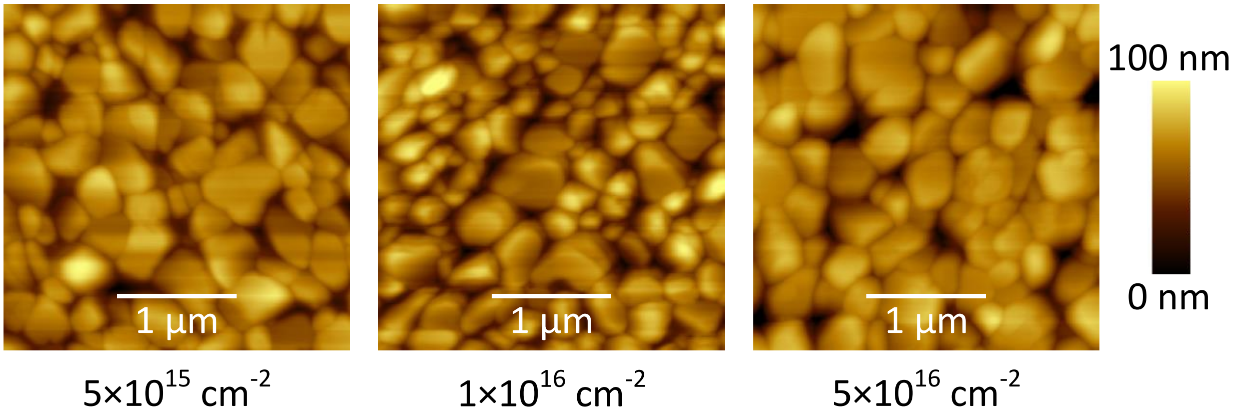

Figure S3. AFM topography of BFO thin films on Ti-implanted Pt/Sapphire substrates.

Figure S3 shows the AFM topographies of BFO thin films on Ti-implanted Pt/Sapphire substrates with a scanning size of 3×3 μm^2. The average grain size of the BFO thin films is around 400 nm, 300 nm and 400 nm for the BFO thin films with Ti fluence of 5×10^{15} cm^{-2}, 1×10^{16} cm^{-2}, and 5×10^{16} cm^{-2}, respectively. The surface roughness of BFO thin films is 12.5 nm, 9.54 nm and 13.1 nm for the Ti fluence of 5×10^{15} cm^{-2}, 1×10^{16} cm^{-2}, and 5×10^{16} cm^{-2}, respectively.

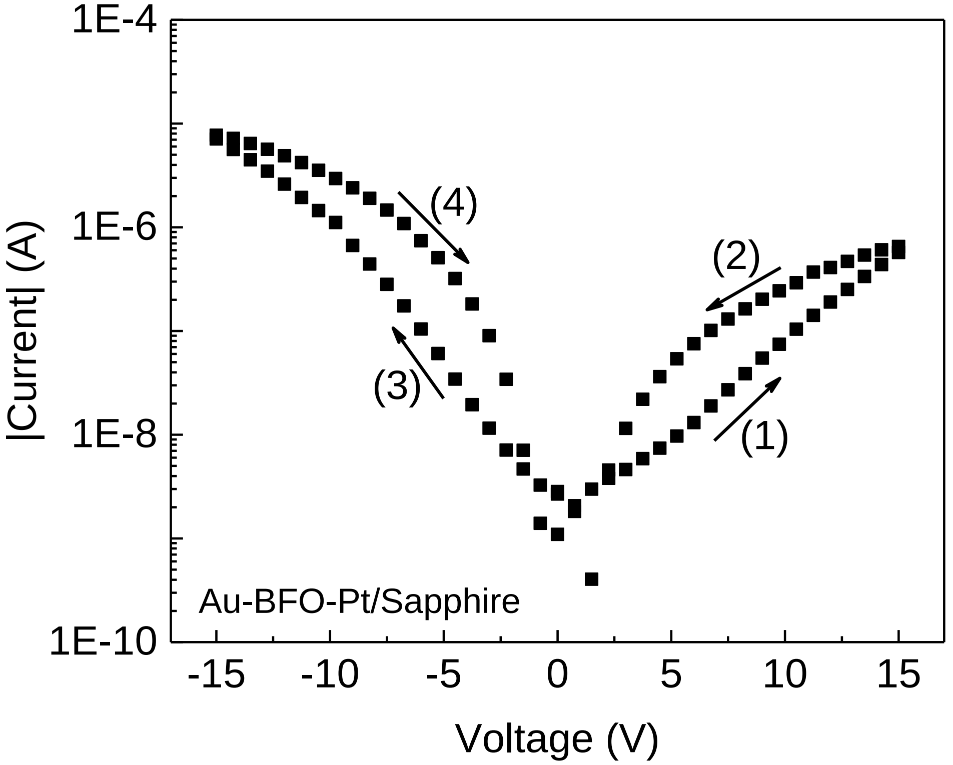

Figure S4. I-V characteristics of BFO thin film deposited on non-implanted Pt/Sapphire substrate

Figure S4 shows the I-V characteristics of BFO thin film deposited on non-implanted Pt/Sapphire substrate. There is no distinct resistive switching behavior observed even when the voltage bias is up to 15 V. The small I-V hysteresis suggests the Schottky barrier heights at top and bottom interface can only be slightly changed by the applied voltage bias.

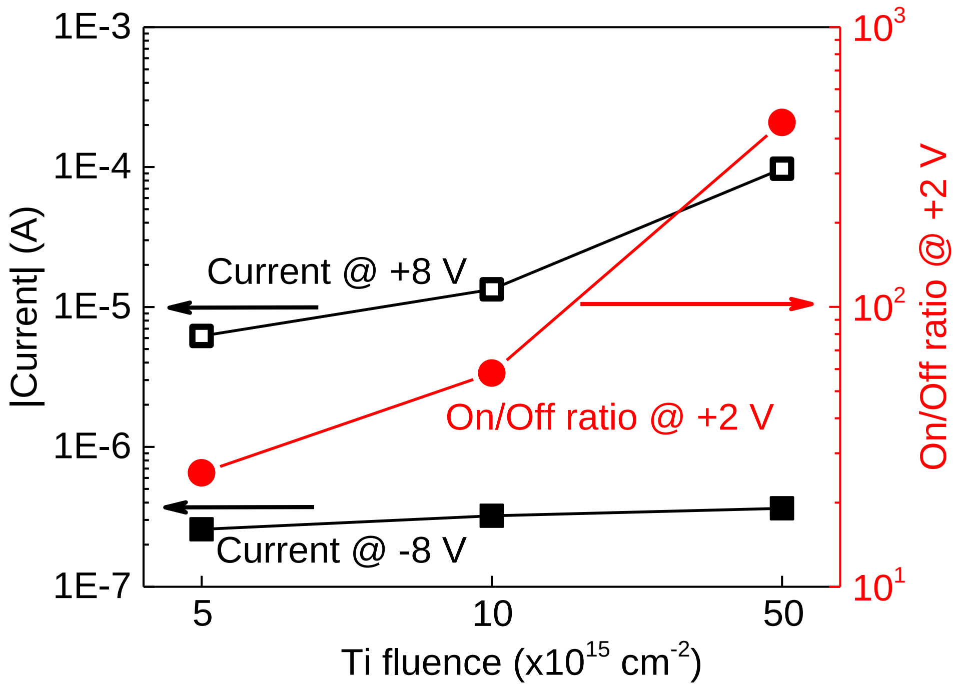

Figure S5. Current at +8 V and -8 V and on/off current ratio at + 2V from the I-V characteristics shown in Figure 2.

Figure S5 shows the current at +8 V and -8 V and the on/off current ratio at +2 V from the I-V characteristics shown in Figure 2. It is clear that the current at +8 V and the on/off current ratio increase with the increasing Ti fluence, while the current at -8 V is nearly constant.

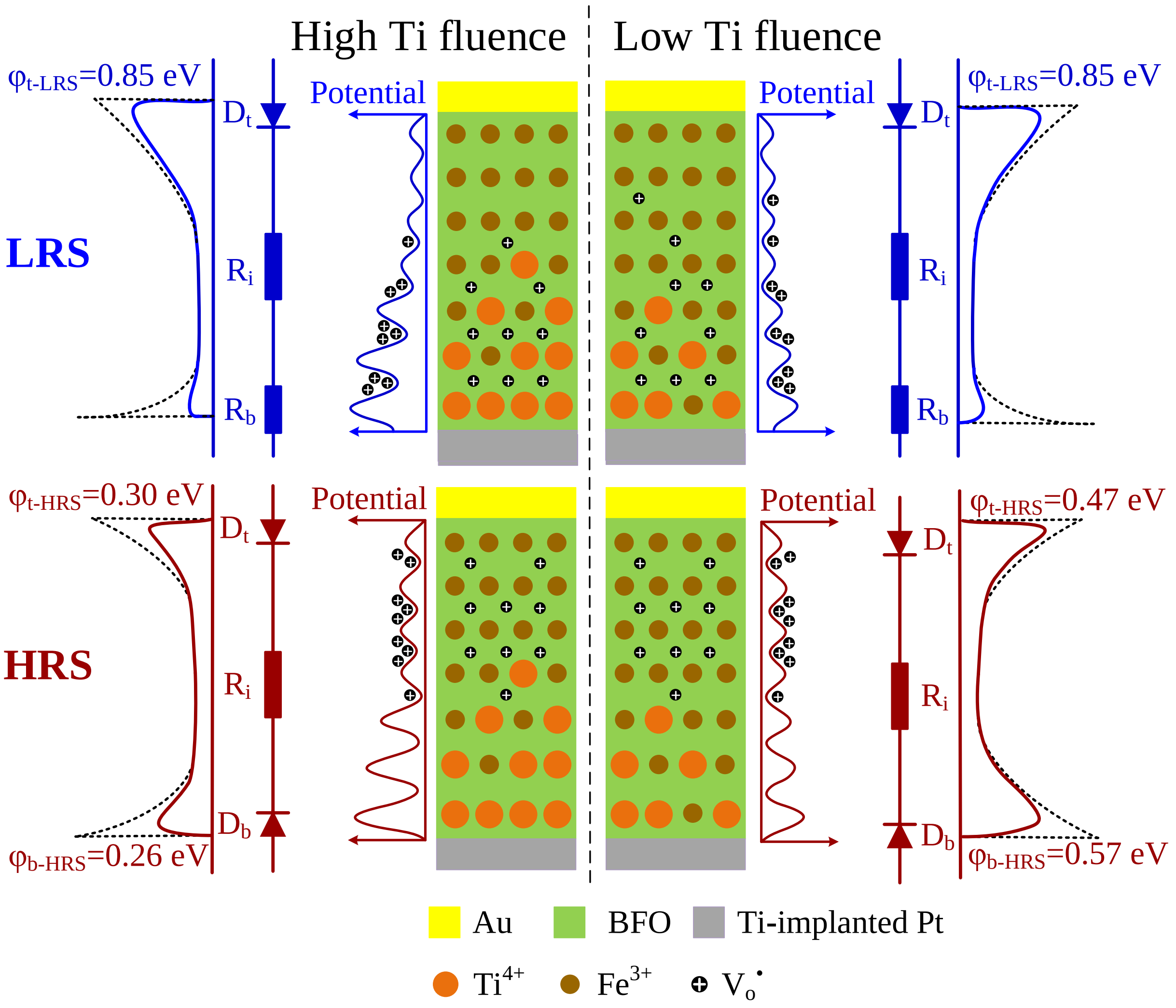

Figure S6. Schematic presentation of the distribution of mobile $V_o^{\bullet}$ (black circles) and fixed Ti^{4+} (orange circles) donors in HRS and LRS for the MIM structures with low/high Ti fluence. The band diagrams of the Schottky barriers, the corresponding equivalent circuits, and schematics of the potential profile for mobile $V_o^{\bullet}$ donors are presented in the left/right side of the schematic for each resistance state. Potential barriers (dashed black lines) which are the potential differences between the work function of BFO and the electrodes. Lowered potential barriers (solid lines) due to image charges.

Figure S6 shows the resistive switching model of a MIM structure with regions with High Ti fluence (left) and Low Ti fluence (right) in LRS (top) and HRS (bottom). In both regions the

Schottky barrier height of the Pt/Ti bottom electrode is modifiable by the drift of mobile oxygen vacancies ($V_o^{\bullet}$) donors under writing bias. The equivalent circuit of HRS is a head-to-head rectifier which consists of two antiserially connected diodes (D_t and D_b) due to the Schottky-like contact ($\varphi_{t\text{-}HRS}$ and $\varphi_{b\text{-}HRS}$) at both top (t) and bottom (b) interface and one resistor R_i denoting the bulk resistance of the BFO thin film. When a positive reading bias is applied, the current is blocked by the reversed bottom diode D_b, thus the MIM structure is in HRS. In Figure 5, the Schottky barrier heights at top and bottom interface ($\varphi_{t\text{-}HRS}$ and $\varphi_{b\text{-}HRS}$) are calculated to be 0.47 eV and 0.57 eV, 0.30 eV and 0.26 eV for the MIM structures with Ti fluence of 5×10^{15} cm^{-2} (Low Ti fluence) and 5×10^{16} cm^{-2} (High Ti fluence), respectively. After applying a positive writing bias, most of the mobile $V_o^{\bullet}$ donors drift towards the BFO-Pt bottom interface, which greatly decreases the bottom Schottky-like barrier height and results in an Ohmic contact (R_b) at the BFO/Ti-implanted Pt bottom interface. By applying a positive reading bias, the diode D_t is forward biased and the MIM structure exhibits LRS. The Schottky barrier height at the Au/BFO top interface ($\varphi_{t\text{-}LRS}$) is around 0.85 eV for all MIM structures in LRS. With enough Ti^{4+} donors (High Ti fluence), several large potential barriers are formed on the atomic scale by the fixed Ti^{4+} donors close to the bottom interface. Once being drifted into the deep potential wells most of the mobile $V_o^{\bullet}$ donors are trapped and can only leave the potential wells within an external electric field. Therefore, the LRS can be well maintained in the MIM structure with high Ti fluence. However, less Ti atoms diffuse into the BFO layer during the PLD process with low Ti fluence, and only some small potential barriers are formed which cannot effectively trap the oxygen vacancies after the positive writing bias. Thus, the LRS is badly maintained.

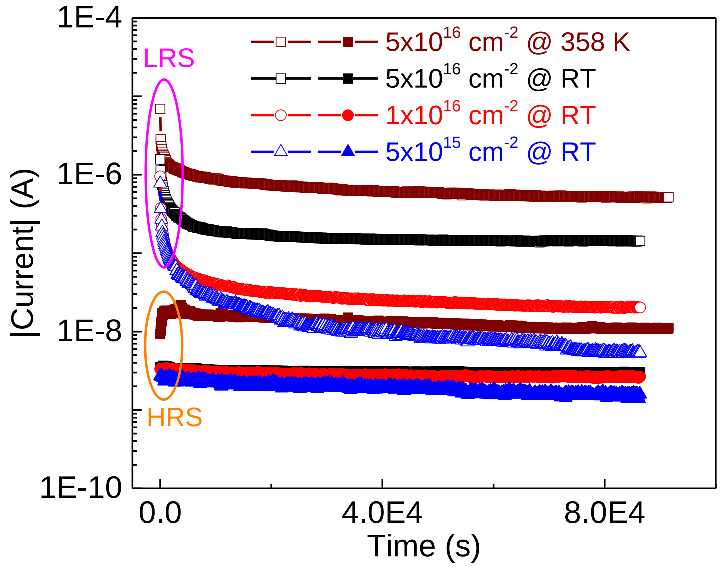

Figure S7. Retention test results on linear time scale.

Figure S7 shows the retention test results on a linear time scale. It is clear that the HRS at room temperature is relatively stable over the retention test process, while LRS show a degradation for all MIM structures. The LRS continuously decrease at room temperature in the MIM structures with Ti fluence of 5×10^{15} cm^{-2} and 1×10^{16} cm^{-2}, and the I_{LRS}/I_{HRS} is smaller than 10 after 24 hours. The LRS of the MIM structure with Ti fluence of 5×10^{16} cm^{-2} initially shows a degradation but becomes stable over within 24 hours at both room temperature and an elevated temperature of 358 K.

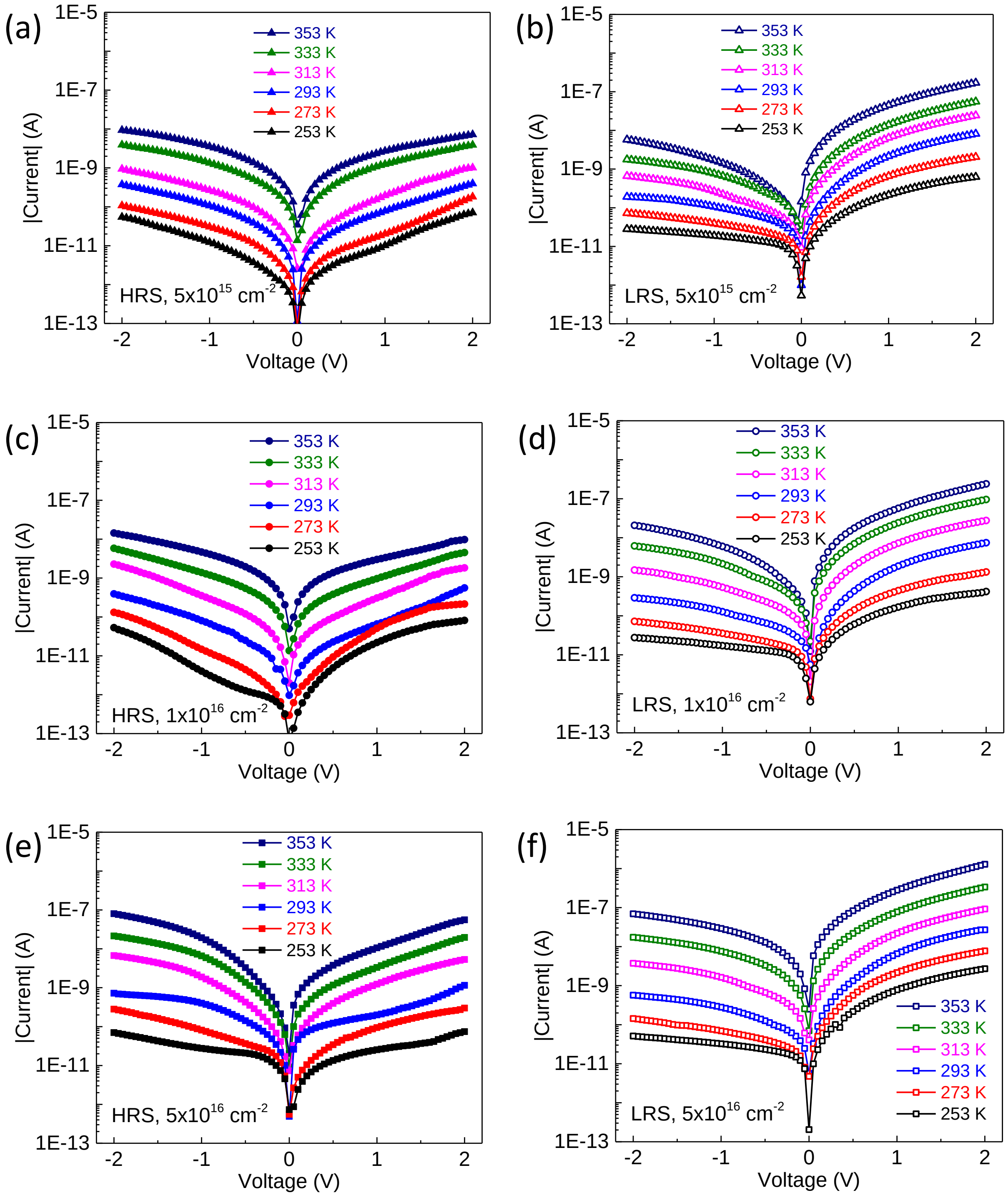

Figure S8. Temperature dependent I-V characteristics for HRS ((a), (c) and (e)) and LRS ((b), (d) and (f)) in MIM structures with Ti fluence of 5×10^{15} cm^{-2} ((a) and (b)), 1×10^{16} cm^{-2} ((c) and (d)), and 5×10^{16} cm^{-2} ((e) and (f)).

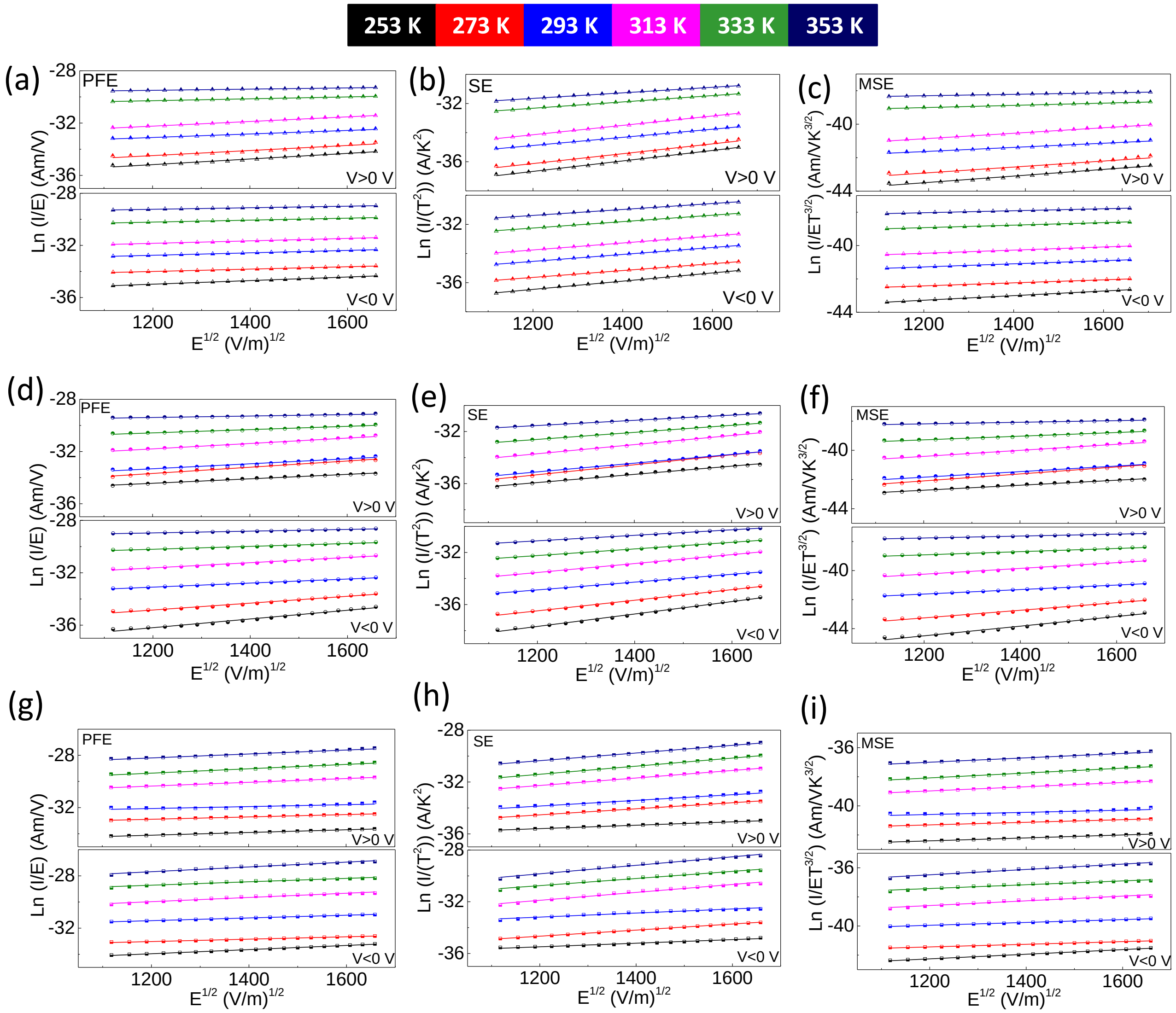

Figure **S9.** Poole-Frenkel emission (PFE), Schottky emission (SE) and modified Schottky emission (MSE) plots for the temperature dependent I-V characteristics of MIM structures in HRS and with Ti implanted bottom electrodes: Ti fluence of 5×10^{15} cm^{-2} (a-c), 1×10^{16} cm^{-2} (d-f), and 5×10^{16} cm^{-2} (g-i).

In HRS, Schottky barriers form at both top and bottom interface and the equivalent circuit is a head-to-head diode. For the temperature dependent I-V characteristics of HRS shown in Figure S8,

the current in positive bias range (V>0 V) and negative bias range (V<0 V) is the reverse current for the bottom and top Schottky diode, respectively. The electric conduction of a reverse biased diode can be governed by the Poole-Frenkel emission (PFE) and Schottky emission (SE) mechanisms.[1] The reverse current dominated by PF emission is given by[1]

$$I \propto E \exp\left(\frac{q}{k_B T}\sqrt{\frac{qE}{\pi\varepsilon_s}}\right) \quad \text{(S1)}$$

whereas in the case of Schottky emission, it is given by[1]

$$I \propto T^2 \exp\left(\frac{q}{2k_B T}\sqrt{\frac{qE}{\pi\varepsilon_s}}\right) \quad \text{(S2)}$$

Simmons showed that the equation S2 is applicable to insulators only if the electronic mean-free path in the insulator is equal to or larger than the thickness of the insulator. For insulators in which the electronic mean-free path is less than the insulator thickness, equation S2 is modified and written as[2]

$$I \propto T^{\frac{3}{2}} E \exp\left(\frac{q}{2k_B T}\sqrt{\frac{qE}{\pi\varepsilon_s}}\right) \quad \text{(S3)}$$

where I is the reverse current, E is the applied electric field, ε_S is the relative dielectric constant, k_B is Plank constant.

Therefore, if the PF emission dominates the reverse current, the plot of $ln(I/E)$ *vs.* $E^{1/2}$ should be linear. Similarly, the linear plots of $ln(I/T^2)$ *vs.* $E^{1/2}$ and $ln(I/ET^{3/2})$ *vs.* $E^{1/2}$ indicate the Schottky emission and the modified Schottky emission contribute the reverse current, respectively. Figure S9 show the plots of $ln(I/E)$, $ln(I/T^2)$, and $ln(I/ET^{3/2})$ as a function of $E^{1/2}$, respectively, for the I-V characteristics in HRS shown in Figure S8. The plots result in linear curves for the PF emission, Schottky emission and modified Schottky emissions. The emission coefficient S can be expressed as follows:

$$S = \frac{q}{nk_B T}\sqrt{\frac{q}{\pi\varepsilon_S}} \quad \text{(S4)}$$

where n=1 for PF emission and n=2 for Schottky emission and n=2 for modified Schottky emission. The coefficients S have been calculated using Equation (S4) and compared with the coefficients obtained by curve fitting in positive bias range (+0.75 V to +1.65 V) and negative bias range (from -0.75 V to -1.65 V) for PFE, SE, and MSE at different temperature are shown in Table SI. Figure S10 shows the ratio of the fitted coefficients and the calculated coefficients. It is clear that only the ratio for the modified Schottky emission is close to 1 at all temperatures for each MIM structure which indicates that the carrier transport in HRS can be described well with the modified Schottky emission.

Table S1 Calculated coefficients and fitted coefficients in both positive and negative bias for PFE, SE, and MSE at different temperatures for the MIM structures with Ti fluence of 5×10^{15} cm^{-2} (I), 1×10^{16} cm^{-2} (II), and 5×10^{16} cm^{-2} (III).

**(I)**	S_{PFE}			S_{SE}			S_{MSE}		
T (K)	Calculated	Fitted (V>0 V)	Fitted (V<0 V)	Calculated	Fitted (V>0 V)	Fitted (V< 0 V)	Calculated	Fitted (V>0 V)	Fitted (V<0 V)
253	0.00070	0.00215	0.00138	0.00139	0.00360	0.00283	0.00139	0.00215	0.00138
273	0.00064	0.00193	0.00091	0.00129	0.00338	0.00235	0.00129	0.00193	0.00091
293	0.00060	0.00134	0.00094	0.00120	0.00279	0.00238	0.00120	0.00134	0.00094
313	0.00056	0.00178	0.00095	0.00112	0.00322	0.00240	0.00112	0.00178	0.00095
333	0.00053	0.00092	0.00076	0.00106	0.00217	0.00220	0.00106	0.00092	0.00076
353	0.00050	0.00076	0.00060	0.00100	0.00191	0.00204	0.00100	0.00076	0.00060

**(II)**	S_{PFE}			S_{SE}			S_{MSE}		
T (K)	Calculated	Fitted (V>0 V)	Fitted (V<0 V)	Calculated	Fitted (V>0 V)	Fitted (V< 0 V)	Calculated	Fitted (V>0 V)	Fitted (V<0 V)
253	0.00070	0.00171	0.00333	0.00139	0.00316	0.00478	0.00139	0.00171	0.00333
273	0.00064	0.00236	0.00262	0.00129	0.00383	0.00407	0.00129	0.00239	0.00262
293	0.00060	0.00191	0.00155	0.00120	0.00336	0.00300	0.00120	0.00191	0.00155
313	0.00056	0.00207	0.00199	0.00112	0.00352	0.00343	0.00112	0.00207	0.00199
333	0.00053	0.00126	0.00110	0.00106	0.00270	0.00255	0.00106	0.00126	0.00110
353	0.00050	0.00085	0.00087	0.00100	0.00200	0.00212	0.00100	0.00085	0.00087

**(III)**	S_{PFE}			S_{SE}			S_{MSE}		
T (K)	Calculated	Fitted (V>0 V)	Fitted (V<0 V)	Calculated	Fitted (V>0 V)	Fitted (V< 0 V)	Calculated	Fitted (V>0 V)	Fitted (V<0 V)
253	0.00070	0.00126	0.00148	0.00139	0.00121	0.00153	0.00139	0.00126	0.00148
273	0.00064	0.00090	0.00091	0.00129	0.00234	0.00236	0.00129	0.00090	0.00091
293	0.00060	0.00094	0.00105	0.00120	0.00219	0.00157	0.00120	0.00094	0.00105
313	0.00056	0.00145	0.00162	0.00112	0.00289	0.00307	0.00112	0.00145	0.00162
333	0.00053	0.00168	0.00131	0.00106	0.00312	0.00276	0.00106	0.00168	0.00131
353	0.00050	0.00151	0.00184	0.00100	0.00296	0.00329	0.00100	0.00151	0.00184

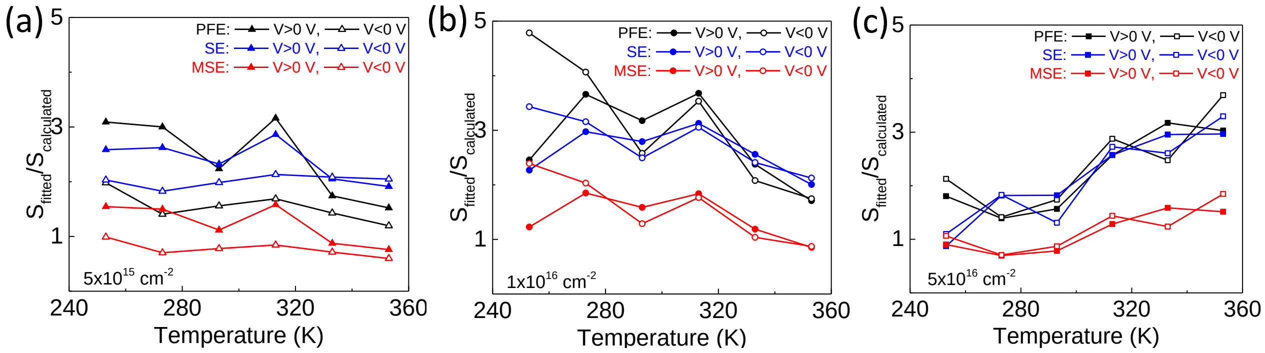

Figure S10. Ratio of the fitted and calculated coefficients for PFE, SE, and MSE at different temperatures for the MIM structures with Ti fluence of 5×10^{15} cm^{-2} (a), 1×10^{16} cm^{-2} (b), and 5×10^{16} cm^{-2} (c).

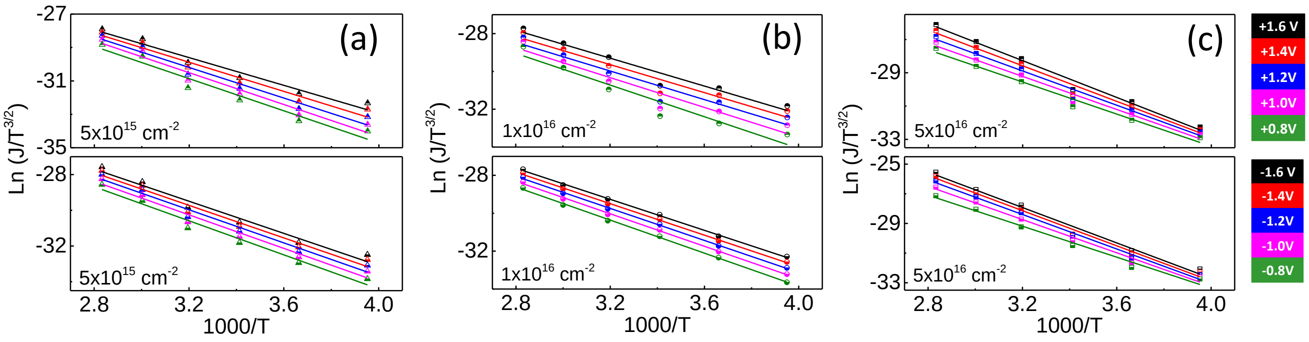

Figure S11. Schottky-Simmons representation in the positive bias range (upper) and negative bias range (lower) for the MIM structures with Ti fluence of 5×10^{15} cm^{-2} (a), 1×10^{16} cm^{-2} (b), and 5×10^{16} cm^{-2} (c) in HRS.

References:

1. Tomer, D., Rajput, S., Hudy, L., Li, C. & Li, L. Carrier transport in reverse-biased graphene/semiconductor Schottky junctions. *Appl. Phys. Lett.* **106**, 173510 (2015).
2. Simmons, J. Richardson-Schottky effect in solids. *Phys. Rev. Lett.* **15**, 967 (1965).
